# The Changes in Concentration of Cerebral Oxygenated Hemoglobin During Single Event-Related Japanese Shiritori Task in Patients With Major Depression Disorder: Comparison With Healthy Subjects

**DOI:** 10.3389/fpsyt.2021.709771

**Published:** 2021-10-14

**Authors:** Youhei Ishii, Yoshihisa Shoji, Mamoru Sato, Shinya Nakano, Akihiko Kondo, Hideya Kodama, Kiichiro Morita

**Affiliations:** ^1^Department of Education and Psychology, Kagoshima Immaculate Heart University, Kagoshima, Japan; ^2^Cognitive and Molecular Research Institute of Brain Diseases, Kurume University, Fukuoka, Japan; ^3^Department of Psychiatry, Kurume University, Fukuoka, Japan; ^4^Nakamura Hospital, Fukuoka, Japan

**Keywords:** near-infrared spectroscopy, single event related design, major depressive disorder, Japanese word production task, cognitive impairment

## Abstract

Patients with major depressive disorder (MDD) have been reported to show cognitive impairments in attention, cognition control, and motivation. The purpose of this study is to compare and examine the characteristics of frontal and temporal cortical activity in outpatients with MDD during the word production task (Shiritori) using a single event-related Near-Infrared Spectroscopy (NIRS) measurement method that was originally devised. The subjects were 29 MDD patients and 29 age matched healthy controls. In this task, one session consisted of two contrasting conditions (word production task, control condition), and all subjects alternated between these conditions. Each word was visually presented by a monitor for 0.3 s as an activation task and a fixed circle was presented for 12 s. In the activation task, subjects had to immediately generate a noun that starts with the last syllable of the presented word and they were required to say only creatures. From the data obtained at each measurement point during the 20 trials, and averaged waveform during activation task (20 trials) was calculated for each channel. During the word production task, the MDD patients showed significantly smaller activation than the controls in the prefrontal cortex area and inferior parietal area, especially in the left area. In addition, there was a significant negative correlation between Δoxy-Hb at the bilateral temporal lobe area and HAM-D total score in the MDD patients. These findings suggest that a single event-related NIRS measurement during Japanese shiritori tasks may be useful tool for evaluating psychophysiological indices in MDD patients, that relationship between activation and symptom may be of help in predicting functional outcome in patients.

## Introduction

Patients with major depressive disorder (MDD) have been reported to show cognitive impairments in executive function, processing speed, attention, and memory (1). The prefrontal cortex plays an important role in the pathology of MDD. Although the definitive pathology of MDD remains unclear, advances in neuroimaging technology have gradually made it possible to identify affected brain regions and networks. Such techniques and methodologies have been proposed as biomarkers for a more reliable diagnosis of psychiatric disorders, including MDD. Under these circumstances, near-infrared spectroscopy (NIRS) has also been reported to be useful as a research tool for various psychiatric disorders including MDD (2, 3). Near-infrared spectroscopy is a method of irradiating the head with near-infrared light absorbed by hemoglobin in the blood, measuring changes in cerebral blood flow in the cerebral cortex by the absorption rate, and indirectly measuring brain activity.

Near-infrared spectroscopy is a relatively new neuroimaging method, was approved in Japan by the Ministry of Health, Labor and Welfare in 2009 as an advanced technique for assisting with the diagnosis of depression. Furthermore, in 2013, NIRS received medical insurance coverage as a supplementary diagnosis. Near-infrared spectroscopy examination has been used clinically as a psychophysiological useful objective indicator reflecting cognitive function.

Near-infrared spectroscopy uses non-invasive light, and has a high temporal resolution (0.1 s) and low spatial resolution (2–3 cm); however, it can be used relatively easily to measure dynamic changes of brain function. The technique makes it possible to visually grasp such changes (4, 5). Changes in the hemoglobin concentration measured by NIRS have been shown to closely correlate with the blood oxygenation level on fMRI (blood oxygenation level-dependent signal) and to be reproducible (6).

Many previous studies using NIRS in MDD patients have generally shown reduced changes in oxy-Hb levels in the frontal lobe region during verbal fluency tasks (VFT) (7). It has been repeatedly reported that depressed patients have less changes in cerebral blood flow in the left prefrontal cortex during VFT than healthy subjects (8, 9). In addition, it is considered that the decrease in left prefrontal cortex function in MDD reflects the loss of interest and especially the symptom of motor rest among the depressive symptoms (10). On the other hand, some NIRS studies on MDD suggest functional decline not only in the left but also in the right prefrontal cortex (11, 12). In a previous using NIRS, Noda et al. (3) investigated the relation between the severity of MDD and frontal lobe activation. It was shown that the right lateral lobe had a significant negative correlation with the total score of the 21-item Hamilton Rating Scale for Depression (HAM-D). This result suggests that the severity of depression affects cognitive function. In fact, Liotti and Mayberg (13) review evidence that strongly suggests the right dorsolateral prefrontal cortex is an important brain structure in emotional/cognitive interactions in negative mood states. Therefore, the severity of depression and depressed mood are closely associated with brain dysfunction and might be observed as an abnormal laterality in depression.

However, many studies using NIRS based on verbal tasks that have been reported to date evaluate brain activity using block design (continue the task for 20–30 s) that intermittently measures blood flow during tasks. However, block design method still have problems with the reproducibility of individual data (14–16). With this block design, it is considered that some patients have difficulty in maintaining attention and concentration on tasks for a long time, and data are likely to vary due to performance and control tasks depending on the stage and condition of the disease. Indeed, Wang et al. (17) reported in a meta-analysis that patients with MDD have impaired sustained attention and may have greater intra-individual variability. Therefore, in this study, we adopted an event-related design to reduce the sustained attention load and overcome performance factors. Therefore, event-related design was adopted in this study. It is a method of calculating event-related blood flow responses obtained by repeating a single task about 20 times or more in order to accurately measure neural activity. It is considered that the variation in performance depending on the patient's condition can be suppressed by repeating a single task, as compared with the task of continuing to perform for a certain period. We hypothesize that we are observing changes in cerebral blood flow based on neurovascular responses. However, it seems to be a change in cerebral blood flow that reflects neural activity with a certain latency.

Furthermore, the “Shiritori” task used in this study is a very popular word chain game generating a word that begins with the last syllable of the preceding word in Japan. In the English-speaking world, it is called “Grab on Behind” or “Last and First.” Since this game is a familiar task that emphasizes the some cognitive functions, including working memory, it is considered to be a task that is easy to teach and easy for many Japanese to understand the rules. In a previous study using block design, Kondo et al. (18) reported that change in blood flow in patients with MDD in the frontotemporal cortices during a similar shiritori task was reduced compared to healthy individuals. In addition, they reported that task performance in the healthy group was higher than in depressed patients, and that there was a correlation between NIRS data and task performance.

Therefore, in this study, the change of oxygen-Hb concentration during the shiritori task was measured by single event-related NIRS measurement in healthy subjects and patients with MDD. The purpose of this study was to explore the usefulness of this measurement method and its potential as a psychophysiological index that reflects the cognitive function of depressed patients.

## Materials and Methods

### Participants

Twenty-nine out-patients with MDD (34.1 ± 7.8 years) and 29 healthy controls (31.0 ± 6.2 years) participated in the study (Table 1). The patients were recruited from the outpatients at Kurume University Hospital, and were diagnosed using ICD-10 criteria by two trained psychiatrists. Almost all patients except for two were medicated with antidepressants. The clinical status of all patients was evaluated by experienced psychiatrists using the HAM-D. All participants were native Japanese speakers and were judged from the Edinburgh Inventory to be right-handed (19). The Japanese version of the National Adult Reading Test assessed their mean intelligence quotient (IQ) values (20). No subjects had a head injury, neurologic disorder, alcohol/substance abuse, epilepsy, visual disabilities, aphasia, or dyslexia.

**Table 1:** Subject characteristics.

**Characteristics**	**Patients** ** (*n* = 29)**	**Controls** ** (*n* = 29)**	**Group difference** ***P*-value**
Age (years: mean± SD)	34.1 ± 7.8	31.0 ± 6.2	0.11
Gender (female/ male)	11/18	13/16	0.59
IQ (JART)	99.3 ± 7.3	103.1 ± 5.7	0.03
Duration of illness (years)	4.2 ± 3.8	NA	NA
Antidepressants (imipramine equivalents) (mg/day)	92.5 ± 79.2	NA	NA
HAM-D total score	15.6 ± 4.5	NA	NA

*JART, Japanese Adult Reading Test; HAM-D, Hamilton Rating Scale for Depression; NA, no applicable*.

Permission for the study was obtained from the ethics committee of Kurume University. After completed description of study, written informed consent was obtained from all participants.

### Procedure

#### NIRS Measurement

**Figure 1:**
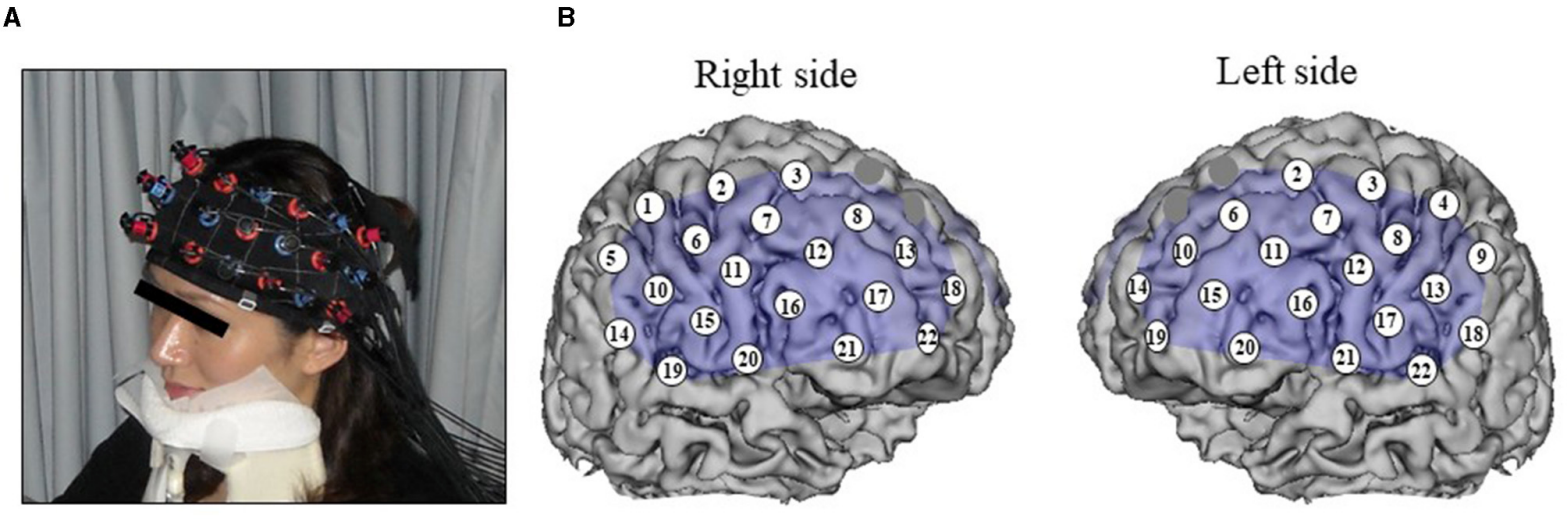
Measurement point of 44 channels for NIRS. **(A)** Jaw fixation in order to eliminate influence of head movement. **(B)** Spatial position of the channels arranged of magnetic resonance imaging.

A 44-channel NIRS system (ETG4000; Hitachi, Tokyo, Japan) measured oxy-Hb changes during tasks covering from the frontal to temporoparietal regions as a recording unit at a sampling frequency of 10 Hz. Oxy-Hb changes were calculated from the difference in absorbance based on the modified Beer–Lambert law. The middle point of the injector–detector probe pairs was defined as a channel. The depth of each channel is supposed to measure changes at points 2–3 cm from the scalp that correspond to the cerebral cortical surface. According to the International 10–20 system used in electroencephalography, we placed probes along the Fp1–Fp2 line to the lowest anterior probes; left channel 19 and right channel 22 (Figure 1). To avoid movement artifacts, participants were instructed to minimize their movements and jaw fixation during examination. The pre-task baseline was determined as the mean during 1 s preceding the word presented, while the post-task baseline was determined as the mean during 1 s from 10 to 11 s after the word was presented. Linear fitting was applied to the data between these two baselines. Next, an averaged waveform for oxy-Hb concentration changes was created, and an area approximation value obtained by analyzing every 100 ms was used as an analysis target (Δoxy-Hb). For the relationship between each channel and anatomic region, NIRS data were converted to a normalized brain image template (three-dimensional composition indication unit; Hitachi).

#### Task Design

**Figure 2:**
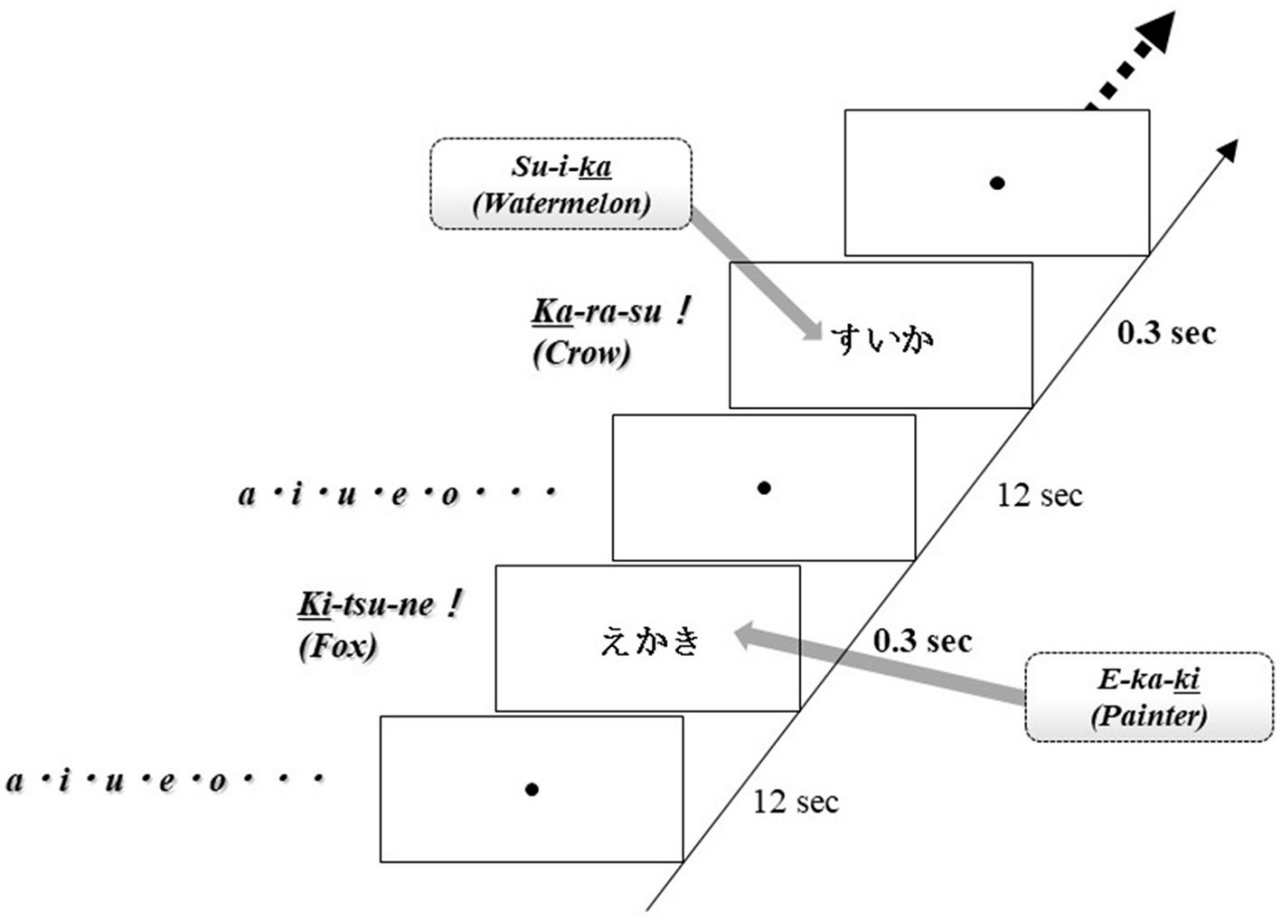
Task design. One session comprised two contrasting conditions (word production tasks, control conditions), and all subjects alternated between these conditions. Each word was visually displayed on the screen for 0.3 s as an activation task and then a fixed circle was displayed for 12 s. In the activation task, subjects were instructed to say a noun immediately starting with the last kana character of the displayed word. The word production task was repeated 20–25 times per session.

Brain activation was measured during word production. The advantage of NIRS is that it is relatively insensitive to body movements during measurement; therefore, this apparatus can obtain data in an overt task. For this examination, each subject sat on a comfortable chair and was required to perform word production. One session consisted of two contrasting conditions (word production task, control condition), and all subjects alternated between these conditions. Each word was visually presented by a monitor for 0.3 s as an activation task and a fixed circle was presented for 12 s. In the activation task, subjects had to immediately generate a noun that starts with the last kana character of the presented word and they were required to say only animal nouns. Thus, this task was the animal category version of the Japanese shiritori word game, as well as a word production task. For example, when the noun “SU-I-KA” (watermelon) was presented, the subject said the noun “KA-RA-SU” (crow). In the control condition, subjects were required to say the syllables “A-I-U-E-O” repeatedly. The word production task was repeated 20–25 times per session (Figure 2).

#### Statistical Analysis

In order to reduce the variability of each reaction, a single task was performed up to 25 times, and the data was analyzed for the 20 times averaged waveforms that could be performed correctly. In addition, the data used for the analysis was limited to the data of subjects with a correct answer rate of 80% or more. In other words, subject data that could not answer correctly at least 20 times by 25 times of stimulation were excluded (note that in this study, all subjects were able to answer correctly by 80% or more). Raw NIRS data were preprocessed by applying a low pass filter with cutoff frequencies of 0.5 Hz. Strangman et al. (21) reported that oxy-Hb changes correlate more strongly with blood-oxygen-level dependent (BOLD) functional MRI signal than do deoxygenated hemoglobin changes; therefore, we adopted the oxy-Hb changes as activation data. We used histograms at each channel to confirm a normal distribution. Profile comparisons between the healthy and patient groups were performed using the unpaired *t*-test except for data on the subject's gender (χ^2^). In the comparison between groups of Δoxy-Hb, due to the nature of the data, the variability was large and the normality could not be confirmed by the Shapiro-wilk test, so the Mann-Whitney U-test, which is a non-parametric method, was performed. For the relationship between Δoxy-Hb and HAM-D total score, Spearman's rank correlation coefficient was calculated for each channel. For analyzing cortical activation, we adopted the false discovery rate correction method [Benjamini and Hockberg method (22)] and set the value specifying the maximum false discovery rate to 0.05. Stat View. 5.0 (SAS) was used as statistical software.

## Results

### Oxy-Hb Changes During Shiritori Task

**Figure 3:**
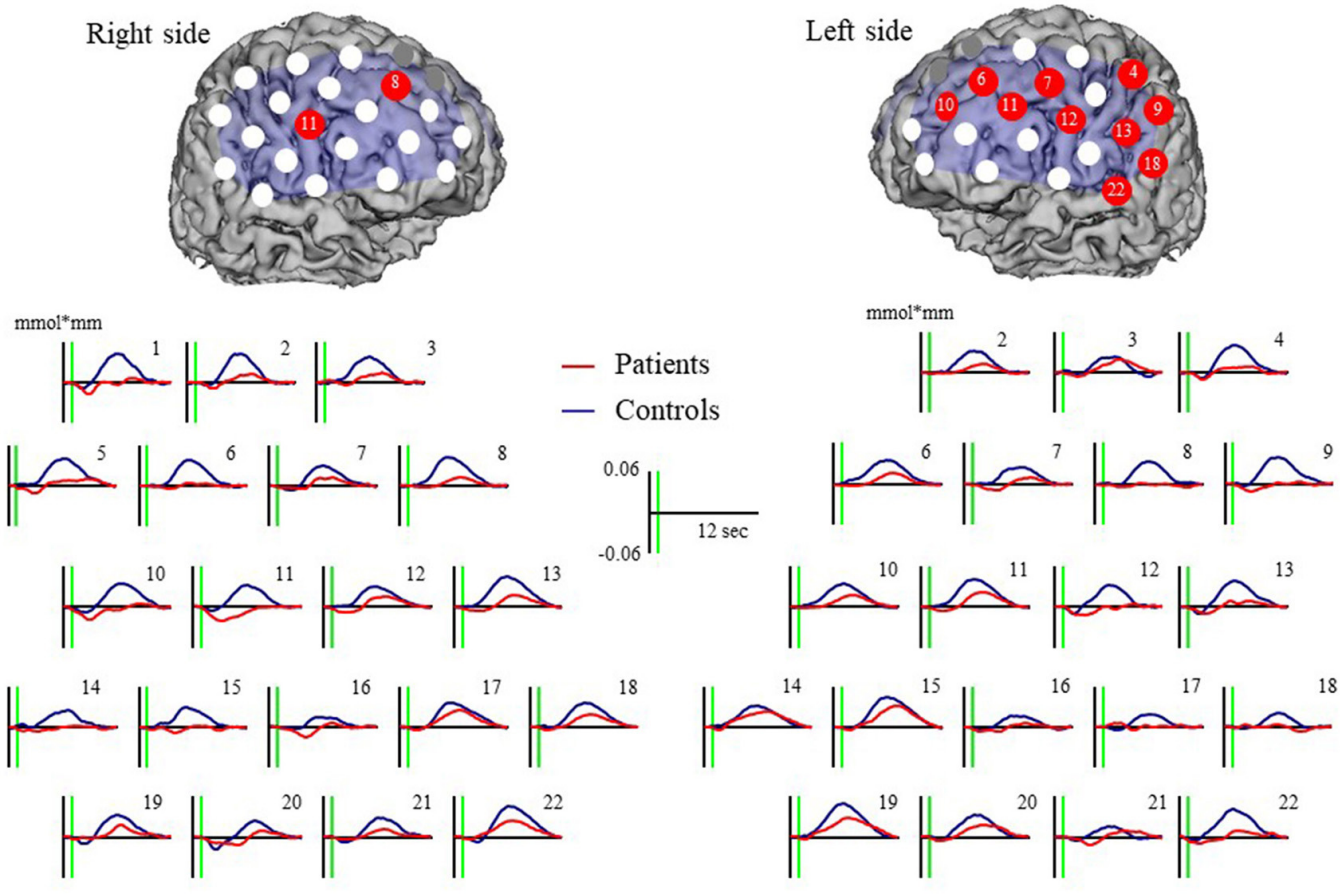
Oxy-Hb grand mean waveforms and channels both with patient and control groups. Red dots indicate channels that significant difference between groups.

Major depressive disorder patients were associated with a significantly smaller increase in oxy-Hb than controls at 12 channels (left ch.4: *U* = 231.00, *p* = 0.0032; left ch.6: *U* = 240.00, *p* = 0.0050; left ch.7: 256.00, *p* = 0.0105; left ch.9: *U* = 175.00, *p* = 0.0001; left ch.10: *U* = 227.00, *p* = 0.0026; left ch.11: *U* = 217.00, *p* = 0.0016; left ch.12: *U* = 216.00, *p* = 0.0024; left ch.13: *U* = 244.00, *p* = 0.0061; left ch.18: *U* = 264.00, *p* = 0.0149; left ch.22: *U* = 239.00, *p* = 0.0077; right ch.8: *U* = 209.00, *p* = 0.0010; and right ch.11 *U* = 208.00, *p* = 0.0016). Waveforms are shown in Figure 3.

### Correlation Between Oxy-Hb Changes and Clinical Variables

**Figure 4:**
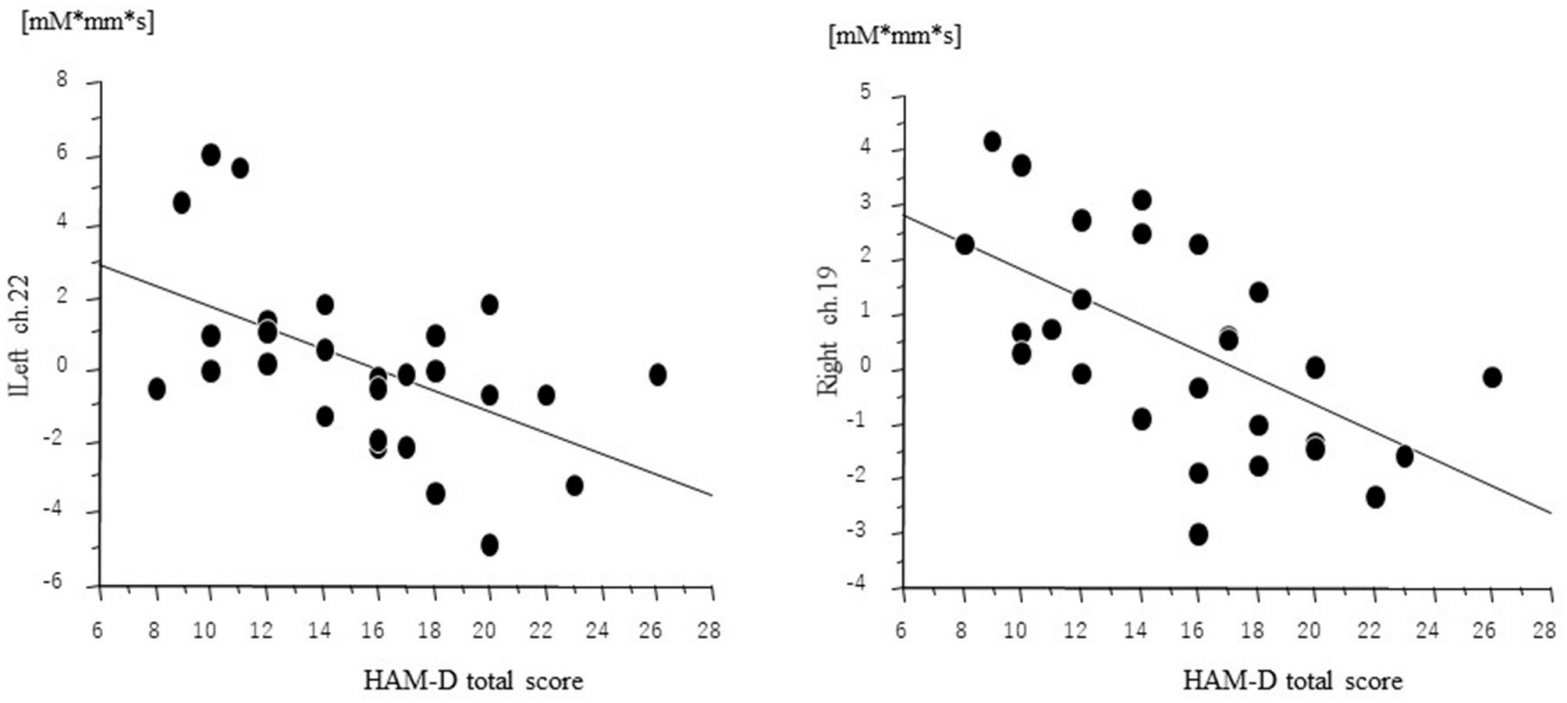
Relation among oxygenated hemoglobin changes (Δoxy-Hb) and HAM-D total score.

Δoxy-Hb revealed a significant negative correlation with HAM-D score at left ch.22 (*r* = −0.55, *p* = 0.0033) and right ch.19 (*r* = −0.59, *p* = 0.0013) for patients with MDD (Figure 4). However, Δoxy-Hb in neither channel was significantly correlated with imipramine equivalents and duration of illness.

## Discussion

In this study, we compared the hemodynamic changes of MDD patients and healthy subjects during the Shiritori task using the single event-related NIRS measurement method. Furthermore, we investigated the relationship between the patient's local cerebral blood flow and the HAM-D total score.

The previous study suggested that NIRS measurement using an event-related method enables researchers to obtain more efficient averaging and to be free from artifacts (23). In the Shiritori task, attention must be paid to the task, the last syllable must be extracted from the word, temporarily held, and then the word with the beginning syllable that matches the last syllable must be searched from the memory, corrected, and answered. This task is associated with the characteristics of both letter and categorical verbal fluency. That is, it requires a wide range of cognitive abilities such as attention, retrival, and working memory. Inoue et al. (24) reported that the dorsolateral prefrontal cortex was activated in the shiritori task in a study using functional MRI. In addition, Yamamoto et al. (25) reported that the shiritori task is suitable for elucidating the brain network related to language in studies using magnetoencephalography. Thus, we thought that the shiritori task of performing overt word production would enable the evaluation of psychiatric patients.

In this study, Δoxy-Hb in the MDD group was significantly lower in the bilateral mid-frontal region, the parietal association region and the left temporal region than in the healthy group, indicating low activation of PFC in patients with MDD as in the previous study. In particular, a decrease in activity was observed in a wide range in the left region. Previous studies using VFT reported that depressed patients had less blood flow changes in the left prefrontal cortex than the controls (8, 9). Such left prefrontal cortex function has also been confirmed by other neuroimaging methods such as fMRI, this is thought to be because VFT is associated with left DLPFC dysfunction compared to other prefrontal cortex function tests (26). A number of previous studies demonstrated increased cortical activity in a depressed group using an n-back working memory task and some demonstrated a higher linear load response in patients with MDD than the normal controls, indicating that hyperfrontality in MDD was more evident in higher cognitive demanding condition. On the other hand, using Tower of London task, Elliott et al. (27) showed reduced neural response in cortical regions, particularly in VLPFC and DLPFC for patients with MDD compared with healthy controls, where patients' performance was impaired. From these previous studies, it is considered that the cerebral hemodynamic response is greatly affected by the difference in task load level and performance. The performance impact was minimized in this experimental design. However, the relationship between neural responses and cognitive demands may cause differences from previous findings using other cognitive tasks. In the future, it will be necessary to make a comparative study that takes into account the level of cognitive demand for issues.

Furthermore, in this study, the MDD group showed significantly lower values than the healthy group not only in the frontal region but also in the parietal association region. The parietal association region is also an area related to higher brain functions such as attention control linked to prefrontal cortex function (28). In working memory tasks such as VFT, previous studies have shown that the phonological loop is associated with the left parietal association region and the central execution system is associated with the prefrontal cortex region (29). Therefore, in the Shiritori task, it is possible that the PFC function is involved in the word generation task and the parietal association region is evoked by phonological working memory. The low activity of the parietal association region in the patient group of this finding may be due to not only dysfunction of the frontal region in MDD, but also a deficiency of the control network with the parietal association region that functionally binds to it.

Regarding the correlation analysis between clinical symptoms and activation values, a significant negative correlation was shown between the bilateral temporal regions and the HAM-D score. This is consistent with previous findings that left prefrontal cortex dysfunction reflects depressive symptoms. In the largest meta-analysis of neuroanatomical differences in MDD using previous cortical thickness measurements, it found that MDD was associated with cortical thinning in the insula, anterior and posterior cingulate, and temporal gyri (30): areas key in salience (31), internal mentation (32), and switching between internal thought and executive control (33). It is not clear whether these anatomical and functional defects are specific or state markers in MDD. However, previous longitudinal studies have found a negative correlation between changes in activation and changes in depressive symptoms in the temporal region (34). According to a study examining the effects of MDD on workplace productivity in eight different countries, the impact of MDD in the workplace is considerable across all countries, both in absolute monetary terms and in relation to proportion of country GDP (35). The MDD can influence the performance of workers who are “present” at work, i.e., presenteeism. Previous research suggests that presenteeism accounts for the majority of the costs (36, 37). This may be more severely impacted by cognitive impairments associated with MDD (38), there is a need to establish an objective index of rehabilitation including cognitive function in MDD patients, in the future, it will be necessary to carry out a longitudinal study that takes into consideration the dosage and duration of illness.

The present research involved several limitations. The first was the size of samples. In this study, we reported the correlation between oxy-Hb changes and HAM-D total score. However, since the size of samples was small, we could not assess the relevance to subordinate items, such as that conducted by previous report (39). Second, all of the patients were on medication. Previous studies reported no change in the oxy-Hb concentration in the PFC during VFT before and after the administration of drug in MDD (2, 40). In our study, the administration of drug in patients was lower, while the duration of illness was longer than in previous studies. This suggests that the decrease in cerebral blood flow variability at temporal region might be a trait marker in patients with MDD, even though the symptoms are stable. Third, there are methodological problems such as the relative value of NIRS data, low spatial resolution, and the possibility of being affected by other than cerebral blood flow such as skin blood flow. Compared to these methodological restrictions, the NIRS system is a methodology that has many advantages such as high time resolution and excellent portability. Also, regarding the handling of data after examination, the fact that the measured data can be immediately converted into a two-dimensional image and fed back to the subject will be a great advantage for clinical application.

In conclusion, NIRS measurement with Single event related design might be a useful psychophysiological method in patients with MDD.

## Data Availability Statement

The raw data supporting the conclusions of this article will be made available by the authors, without undue reservation.

## Ethics Statement

The studies involving human participants were reviewed and approved by the Ethics Committee of Kurume University. The patients/participants provided their written informed consent to participate in this study. Written informed consent was obtained from the individual(s) for the publication of any potentially identifiable images or data included in this article.

## Author Contributions

KM: conception and design of the study. YI and YS: analysis and interception of data. YI, AK, and SN: collection and assembly of data. YI: drafting of the article. YS, MS, and HK: critical revision of the article for important intellectual content. YS and KM: final approval of the article. All authors contributed to the article and approved the submitted version.

## Funding

This research was partly supported by a Grant-in-Aid for Scientific Research from the Japan Society for the Promotion of Science (23591734).

## Conflict of Interest

The authors declare that the research was conducted in the absence of any commercial or financial relationships that could be construed as a potential conflict of interest.

## Publisher's Note

All claims expressed in this article are solely those of the authors and do not necessarily represent those of their affiliated organizations, or those of the publisher, the editors and the reviewers. Any product that may be evaluated in this article, or claim that may be made by its manufacturer, is not guaranteed or endorsed by the publisher.
